# ﻿Two new species of genus *Labronema* Thorne, 1939 (Nematoda, Dorylaimidae) from natural parks of Vietnam with an identification key to the species with a medium-sized odontostyle

**DOI:** 10.3897/zookeys.1215.128183

**Published:** 2024-10-11

**Authors:** Tam T. T. Vu, Milka Elshishka, Anh D. Nguyen, Thi Mai Linh Le, Aleksandar Mladenov, Vlada Peneva

**Affiliations:** 1 Institute of Ecology and Biological Resources, Vietnam Academy of Science and Technology, 18 Hoang Quoc Viet, Cau Giay, Hanoi, Vietnam Institute of Ecology and Biological Resources, Vietnam Academy of Science and Technology Hanoi Vietnam; 2 Graduate University of Science and Technology, Vietnam Academy of Science and Technology, 18 Hoang Quoc Viet, Cau Giay, Hanoi, Vietnam Graduate University of Science and Technology Hanoi Vietnam; 3 Institute of Biodiversity and Ecosystem Research, Bulgarian Academy of Sciences, 2 Gagarin Street, 1113 Sofia, Bulgaria Institute of Biodiversity and Ecosystem Research, Bulgarian Academy of Sciences Sofia Bulgaria

**Keywords:** Distribution, Dorylaimida, morphology, phylogeny, 18S rDNA, 28S rDNA

## Abstract

*Labronema* Thorne, 1939 is a large and diverse dorylaimid genus with complicated taxonomy. Two new species, *Labronemaporosum***sp. nov.** and *Labronemabidoupense***sp. nov.** from natural habitats in Vietnam were characterised both morphologically and molecularly (18S rDNA and 28 rDNA), and line drawings and microphotographs are provided. Phylogenetic analyses showed that the new species clustered together with *Labronemaferox* Thorne, 1939, the type species of the genus. The two new taxa belong to a group of *Labronema* species with medium body (L = 1.5–2.5 mm) and odontostyle (31–39 μm) length, and a lip region offset by a constriction. Based on morphological and molecular evidence, this study shows that the populations from Vietnam previously identified as *L.glandosum* Rahman, Jairajpuri, Ahmad & Ahmad, 1986 in fact represent *L.porosum***sp. nov.** Evolutionary relationships of *Labronema* species are discussed. A key to the species of *Labronema* with a medium-sized odontostyle (31–39 μm) is provided as well as a list of the species of the genus considered valid.

## ﻿Introduction

The genus *Labronema* was erected by Thorne (1939) when he described three new species and transferred six other species of the genus *Dorylaimus* Dujardin, 1845, with *Labronemaferox* Thorne, 1939 designated as a type species. Siddiqi (1969) raised the subfamily Qudsianematinae Jairajpuri, 1965 containing three genera including *Labronema* Thorne, 1939 to a full family rank. Based on an integrative approach including morphology, postembryonic development, and analysis of D2-D3 expansion segments of 28S rDNA sequences, Peña-Santiago and Álvarez-Ortega (2014) transferred the genus *Labronema* and seven other genera of the family Qudsianematidae to Dorylaimidae and proposed for it a new subfamily Labronematinae. Several studies showed that genus *Labronema* is a heterogeneous group of species with a very complicated taxonomy and systematics (Andrássy 2009; Álvarez-Ortega et al. 2010; Peña-Santiago 2019; Peña-Santiago and Vinciguerra 2019; Peña-Santiago 2022). One of the main problems, similarly to other nematode taxa, is that the descriptions of many species do not entirely fit the genus diagnosis, often they do not conform to the modern taxonomic requirements and are insufficiently detailed (Peña-Santiago and Vinciguerra 2019; Altash et al. 2024; Elshishka et al. 2024). All that does not allow a good species differentiation. Furthermore, approximately 40% of the species (Andrássy 2011a) are described based on one sex, and this does not facilitate elucidating the systematics of the genus. Not less important is the limited molecular data (several 18S and 28S rRNA gene sequences), which are often not supported by morphological characteristics (Peña-Santiago 2019).

For several decades, a number of species, which were identified under *Crassolabium* Yeates, 1967, *Thonus* Thorne, 1974, *Eudorylaimus* Andrássy, 1959, *Aporcelaimellus* Heyns, 1965, *Talanema* Andrássy, 1991 have been transferred to or moved out *Labronema* (Andrássy 1991, 2009, 2011a; Peña-Santiago and Ciobanu 2011; Álvarez-Ortega and Peña-Santiago 2013, Imran et al. 2021). Two species of *Labronema* were transferred to *Nevadanema* Álvarez-Ortega & Peña-Santiago, 2012.

During the last ten years only three new species of the genus have been described: *L.mannai* Dattaray, Roy & Gantait, 2015 and *L.minimus* Dattaray, Roy & Gantait, 2015 from India, and *L.montanum* Peña-Santiago & Abolafia, 2019 from Spain. Furthermore, several studies presented new information on some known species contributing to the improvement of the taxonomy of the genus. Peña-Santiago (2019) provided a redescription of *L.ferox*, the type species of the genus, based on the population studied by Thorne (1974). This detailed study showed that *L.ferox* is a widespread species only in North America. Peña-Santiago and Vinciguerra (2019) redescribed three other species originally described from Italy: *L.angeloi* Vinciguerra & Clausi, 1994, *L.carusoi* Vinciguerra & Orselli, 1998, and *L.pulchrum* Vinciguerra & Zullini, 1980. In their paper, *L.pulchrum* was regarded as a junior synonym of *L.duhouxi* (Altherr, 1963).

Based on the morphological characters (species with a transverse vulva; a conoid, rounded or digitate tail, and non-contiguous ventromedian supplements) and molecular characterisation (D2-D3 expansion segments of 28S rRNA and 18S rRNA gene sequences), Imran et al. (2021) transferred *Labronemabaqrii* Khan, Jairajpuri & Ahmad, 1989 to the genus *Talanema*. The authors regarded *L.neodiversum* Mondal, Manna & Gantait, 2012 as a junior synonym of *T.baqrii*, and transferred three other *Labronema* species (*L.malagasi* Furstenberg, Heyns & Swart, 1993, *L.digiturum* Vinciguerra, 1984, and *L.sphinctum* Mohilal & Dahanachand, 2001) to the genus *Talanema*. Peña-Santiago (2022) synonymized *L.loeffleri* Andrássy, 1978 and *L.macrosoma* Alekseyev, 1992 with *L.magnum* Altherr, 1972 on the base of the following morphological characters: large general size, very strong odontostyle, large spicules, and similar number of ventromedian supplements as well as similar habitats. Recently, Zahedi Asl et al. (2023) presented new integrative data (morphological and molecular) of *Labronemavulvapapillatum* (Meyl, 1954) Loof & Grootaert, 1981 from Iran.

Imran et al. (2021) considered that in terms of the lip region shape, *Labronema* spp. present a continuum of variation comprising (i) *L.ferox* type having offset lip region with arched lips, (ii) *L.vulvapapillatum* type having offset lip region but lips not arched, and (iii) *L.hyalinum* (Thorne & Swanger, 1936) Thorne, 1939 type having lip region almost continuous with adjacent body. The species having atypical lip region were referred to as “atypical” ones (Álvarez-Ortega et al. 2010). However, in order to elucidate the status of these groups more integrative taxonomic studies are needed.

Currently, the genus *Labronema* includes 47 species (see Suppl. material 1), spread in all continents, except for Antarctica. In Vietnam, two species of the genus *Labronema*, *L.neopacificum* Rahman, Jairajpuri, Ahmad & Ahmad, 1986 and *L.glandosum* Rahman, Jairajpuri, Ahmad & Ahmad, 1986 have been reported (Álvarez-Ortega et al. 2010; Vu and Nguyen 2013). During an extensive study of the free-living nematodes of protected territories in Vietnam, two unknown species of *Labronema* were recovered. The objective of the present study is (i) to characterize these populations on the basis of an integrative approach and describe the new species, (ii) to discuss their phylogenetic relationships and (iii) to provide an identification key to the species with a medium-sized odontostyle.

## ﻿Materials and methods

### ﻿Nematode extraction, preservation, and morphological studies

Soil samples were collected from a pristine forest in the Natural Reserve Du Gia (Bac Me District, Ha Giang Province) and the National Park Bidoup-Nui Ba (Lac Duong District, Lam Dong Province), Vietnam. Nematodes were extracted from soil samples using modified Baermann funnel technique (Southey 1986). They were heat-killed, fixed in TAF solution (for morphological observations) or in a DESS mixture (Yoder et al. 2006) (for molecular analyses), transferred to anhydrous glycerol (Seinhorst 1962), and mounted on glass slides for microscopic observation. Drawings were prepared using an Olympus BX 51 compound microscope with a drawing tube. Photomicrographs were taken using an Axio Imager.M2-Carl Zeiss compound microscope equipped with a digital camera (ProgRes C7) and specialised software (CapturePro Software 2.8). Measurements were made using an Olympus BX 41 light microscope with a drawing tube and digitising tablet (CalComp Drawing Board III, GTCO CalCom Peripherals, Scottsdale, AZ, USA) and Digitrak 1.0f computer program (Philip Smith, John Hutton Institute, Dundee, UK). Terminology follows Peña-Santiago (2021). The locations of pharyngeal gland nuclei are given following Loof and Coomans (1970).

### ﻿DNA isolation, amplification, and sequencing

Genomic DNA was isolated from single individuals as described by Holterman et al. (2006) and stored at –20° until used as a PCR template. The D2-D3 expansion segment of 28S and was amplified using the forward D2A (5′–ACAAGTACCGTGGGGAAAGTTG–3′) and reverse D3B (5′–TCGG AAGGAACCAGCTACTA–3′) primers (Subotin et al. 2006) and the 18S rDNA fragment was amplified using the primers 18S (18F: 5’-TCTAGAGCTAATACATGCAC-3’/18R: 5’-TACGGAAACCTTGTTACGAC-3’) (Floyd et al. 2005). All PCR reactions contained 12.5 μl Hot start green PCR Master Mix (2×) (Promega, USA), 1 μl of the forward and reverse primer (10 μM each), the 3 μl DNA template and sterile Milli-Q water to 25 μl of the total volume. All PCR reactions were performed in SimpliAmp Thermal cycler (Thermo Fisher Scientific) as follows: an initial denaturation step at 95 °C for 4 min, followed by 40 cycles at 95 °C for 30 s, 54 °C for 30 s and 72 °C for 60s with a final incubation for 5 min at 72 °C. Amplicons were visualised under UV illumination after Simplisafe gel staining and gel electrophoresis.

### ﻿Phylogenetic analyses

For reconstructing the phylogenetic relationships, analyses were based on 18S and 28S rDNA sequences. The newly obtained rDNA sequences were analysed using the BioEdit and aligned with sequences available in GenBank using the ClustalW alignment tool implemented in the MEGA 7 version 11.0 (Kumar et al. 2016). The final 18S and 28S rDNA datasets for phylogenetic study included sequences from the present study and available sequences of members of the Dorylaimidae retrieved from GenBank. The prepared multiple alignments of 28S rDNA generated by the ClustalW algorithm were routinely manually edited in order to eliminate improper phylogenetic signals. Representative *Mononchus* sequences were used as the outgroup. The phylogenies were constructed with the MEGA 7 version 11.0. Maximum likelihood with K2+G+I substitution model for 28S; T92+G+I substitution model for 18S data set was used. Genetic distances (number of nucleotide positions and uncorrected p-distance) were calculated in MEGA7.

## ﻿Taxon treatment

### ﻿ Labronema porosum sp. nov.

Taxon classificationAnimaliaDorylaimidaDorylaimidae

C080CB1A-6C3D-56C3-92BD-04E5B29E615D

https://zoobank.org/8C545ECD-4517-47DB-BD84-43D2A9F1DD28

Labronema glandosum sensu Vu and Nguyen (2013).

#### Material examined.

Five females from the Natural Reserve Du Gia (Bac Me District, Ha Giang Province) in good condition.

#### Description.

**Figure 1.**
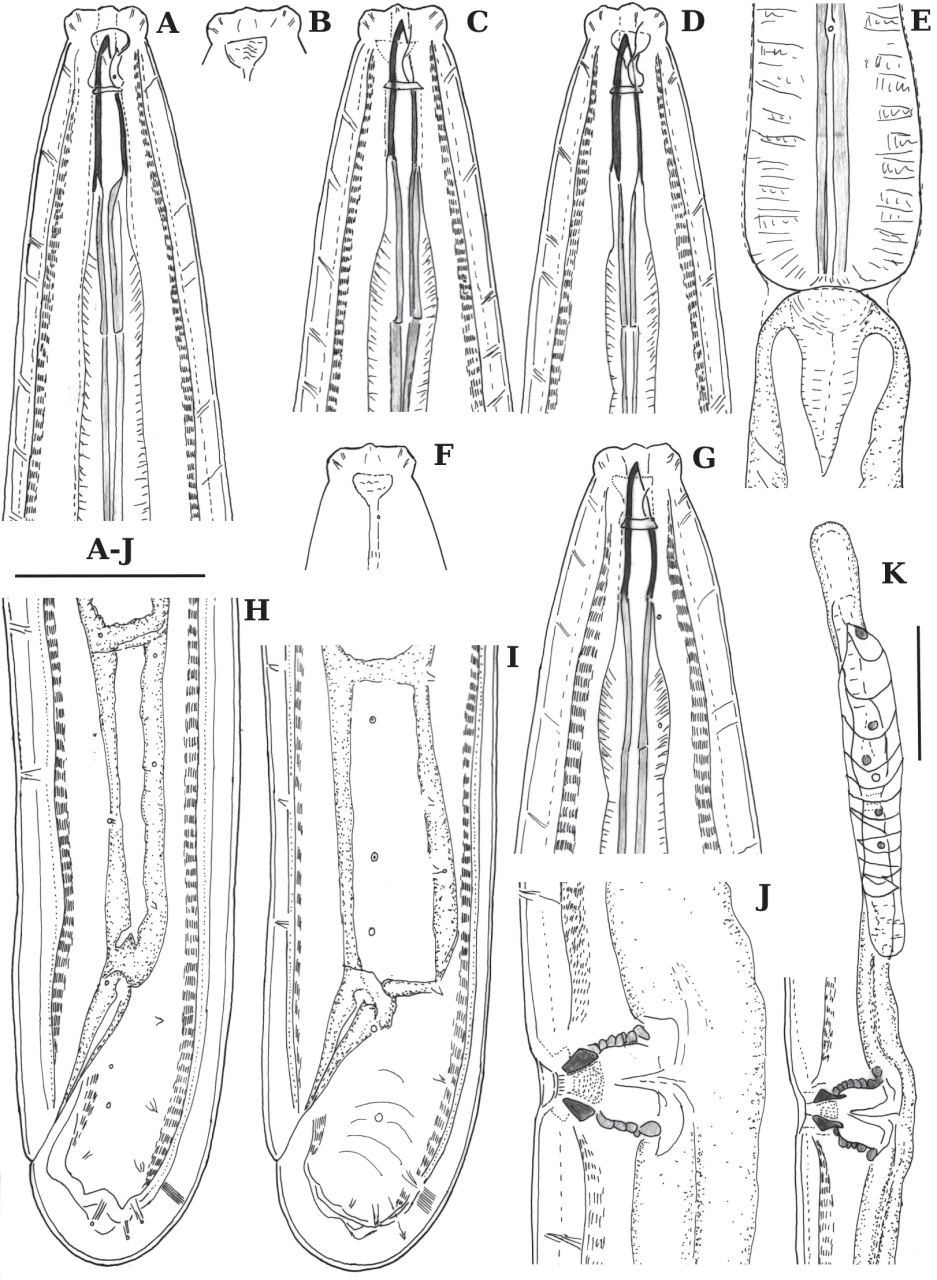
Line drawings of *Labronemaporosum* sp. nov. female **A, C, D, G** lip region (C holotype) **B, F** amphids (B holotype) **E** pharyngo-intestinal junction (holotype) **H, I** tail ends (H holotype) **J** vulval region **K** anterior genital branch (holotype). Scale bars: 25 µm (**A–J**); 50 µm (**K**).

**Figure 2.**
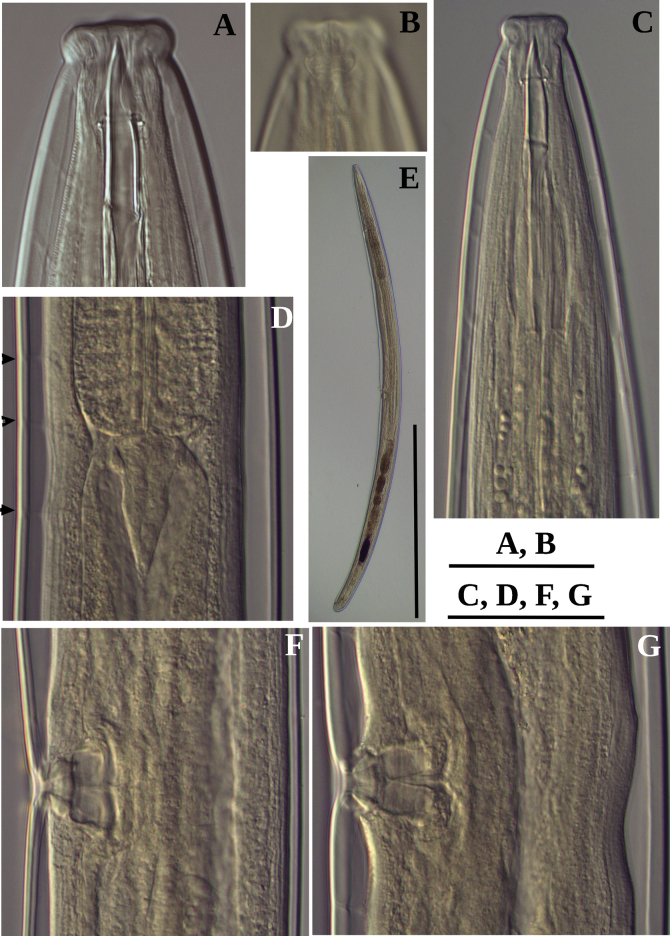
Photomicrographs of *Labronemaporosum* sp. nov. females **A** lip region **B** amphidial fovea **C** anterior end **D** pharyngo-intestinal junction, arrows indicate dorsal pores **E** entire body **F, G** vulval region. Scale bars: 30 µm (**A, B**); 50 µm (**C, D, F, G**); 500 µm (**E**).

**Figure 3.**
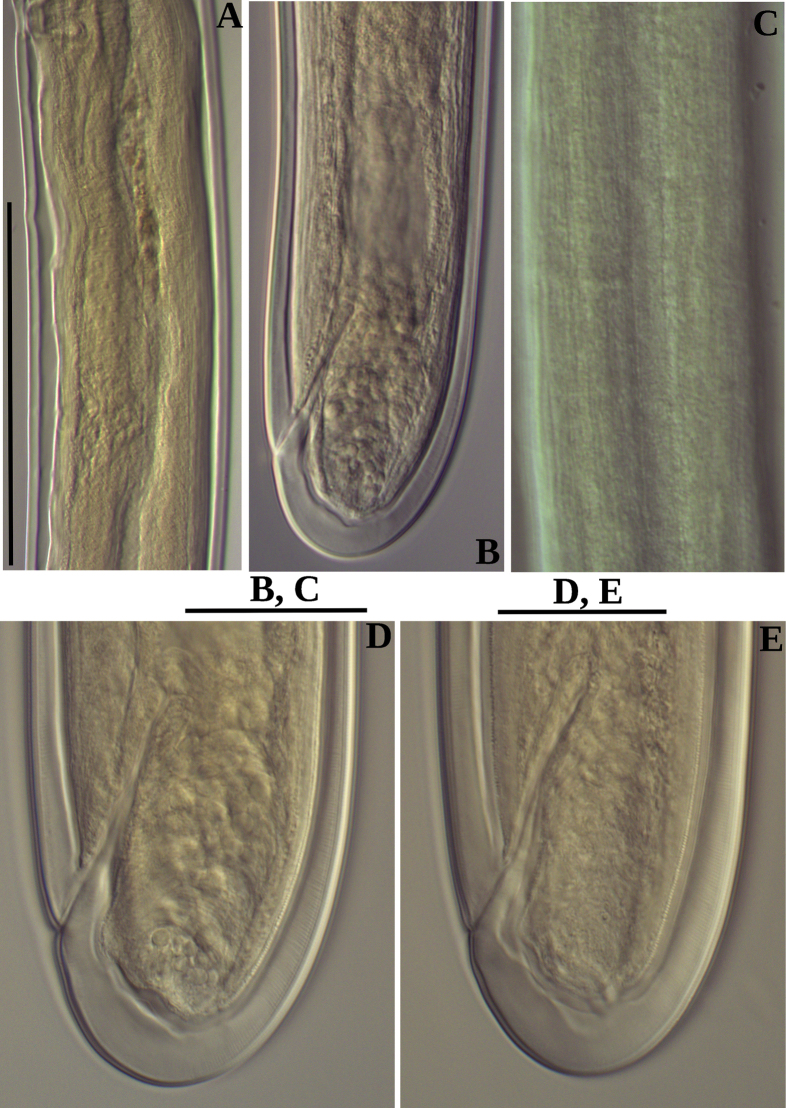
Photomicrographs of *Labronemaporosum* sp. nov. females **A** posterior uterus **B, D, E** tail ends **C** lateral field. Scale bars: 200 µm (**A**); 50 µm (**B, C**); 30 µm (**D, E**).

**Females** (for measurements see Table 1, Figs 1–3) Nematodes of a medium size. Body cylindrical, slightly ventrally curved after fixation. Cuticle three layered, especially obvious at caudal region, outer layer thin, intermediate – much thicker than the outer one, particularly at the caudal region; inner layer much thinner than the intermediate layer, especially distinct at the caudal region; cuticle 4–7 μm thick at anterior region, 5–7 μm at mid-body, and 7–9 μm posterior to anus. The intermediate layer with longitudinal striations seen at a certain optical section. Ventral and lateral pores conspicuous, present along the whole body, between fifth and sixth ventral pore a rudimentary duct was observed (at 95–112 µm from anterior end); dorsal pores 5–7 at anterior end. Lip region truncated, laterally weakly angular, offset by an appreciable constriction, 2.8–3.1 times wider than high, less than one-third (23–28%) of body diameter at pharynx base. Lips separated, outer labial and cephalic papillae very low, inner labial papillae slightly protruding. Amphid with stirrup-shaped fovea; its aperture 9–10.5 μm wide, occupying two-fifths to almost one-half (39–47%) of lip region diameter. Cheilostom nearly cylindrical, with thick walls. A peculiar cuticular fold present on the dorsal site of stoma, its posterior end reaching the guide ring. Odontostyle strong, 5–6 times as long as broad, 1.4–1.6 times longer than lip region diameter, and 1.7–1.9% of total body length. Odontophore rod-like; 1.1–1.2 times odontostyle length. Pharynx conspicuously muscular, with its slender portion enlarging very gradually, the basal expansion 220–237 μm long, 45–47% of pharynx length; dorsal nucleus (DN) at 56–60% of pharyngeal length (*n* = 3). Pharyngo-intestinal junction well developed; cardia with conical projection into the intestinal lumen, measuring 46–59 × 20–25 μm. Prerectum 1.9–2.0 and rectum 1–1.2 times longer than anal body diameter. Genital system di-ovarian, with both branches well and equally developed. Ovaries large, reaching oviduct/uterus junction, with oocytes first in two or more rows and then in a single row, anterior 161–229 μm and posterior ovary 170–249 μm long, respectively. Anterior oviduct 97–180 μm long and posterior 70–120 μm long (*n* = 3), respectively or 1.0–2.1 and 0.7–1.4 times longer than body diameter, *pars dilatata oviductus* weakly developed, sphincter at oviduct/uterus junction 7–8 μm long, not cuticularised. Uteri not differentiated, tubular, sperm not present in them, anterior uterus 114–125 μm long, posterior uterus 95–100 μm long or slightly longer than the body diameter. Uterine egg observed in one female (107 × 53 μm). Vagina extending inwards to 37–45% of the body diameter: *pars proximalis vaginae* measuring 20–24 × 17–20 μm, surrounded by weakly developed musculature, *pars refringens* consisting of two small triangular sclerotized pieces (lateral view), with a combined width of 17–20 μm; *pars distalis* 7.5–9.0 μm long. Vulva a longitudinal slit, 8–10 µm long. Tail short, rounded. Hyaline part of tail 11–17 μm thick or 40–65% of total tail length.

**Table 1.** Measurements of females of *Labronemaporosum* sp. nov. and females and males of *Labronemabidoupense* sp. nov. from Vietnam. All measurements are in μm (except L in mm), and in the form mean ± standard deviation with range.

Character	*L.porosum* sp. nov.		*L.bidoupense* sp. nov.		
Type	holotype	paratypes	holotype	paratypes	
**Number of specimens**	♀	4 ♀♀	♀	5 ♀♀	6 ♂♂
L	1.94	1.95 ± 0.1 (1.92–2.02)	1.59	1.90 ± 0.1 (1.77–2.04)	1.72 ± 0.2 (1.41–1.96)
a	22.5	19.7 ± 2.1 (18.2–21.1)	23.4	22.4 ± 1.0 (20.9–23.3)	22.6 ± 2.2 (19.7–25)
b	3.8	4.0 ± 0.1 (3.9–4.0)	3.6	4.0 ± 0.1 (3.8–4.1)	4.1 ± 0.3 (3.6–4.5)
c	75.4	73.5 ± 5.9 (69.3–77.6)	58.9	69.2 ± 8.2 (57.8–77.2)	62.9 ± 3.1 (58.8–67.8)
c’	0.6	0.5 ± 0.1 (0.5–0.6)	0.6	0.6 ± 0.1 (0.5–0.6)	0.5 ± 0.03 (0.5–0.6)
V%	53	50.9 ± 1.6 (48.5–52)	57	55. 6 ± 1.2 (54–57)	–
Lip region diameter	24	23.4 ± 0.8 (22–24)	26	26.3 ± 0.9 (25–28)	23.9 ± 1.0 (23–26)
Odontostyle length	37	35.9 ± 0.9 (35–37)	39	38.3 ± 0.7 (37.5–39)	34.5 ± 1.0 (33–36)
Odontophore length	42	42.2 ± 2.0 (40–44)	42	43; 43; 43	50.7 ± 1.8 (48–53.5)
Guiding ring	21	20.7 ± 0.4 (20–21)	22	22.5 ± 0.4 (22–23)	20.1 ± 1.3 (18–21)
Neck length	510.5	491.1 ± 8.4 (484–500)	443	470.9 ± 11.9 (458–487)	422.9 ± 22.8 (388.5–450)
Body diameter at:					
- Pharynx base	85	93.7 ± 10.2 (82.5–103)	68	78.3 ± 2.4 (76–81)	71.7 ± 7.5 (57–77)
- Mid - body	86	101.5 ± 8.3 (91–111)	68	84.9 ± 3.5 (80.5–90)	76.7 ± 10.9 (59–89.5)
- Anus/cloacal aperture	45	51.4 ± 4.9 (48–55)	43	50.3 ± 2.4 (47–53)	50.9 ± 3.6 (45–55)
Distance vulva to anterior end	1030	992.8 ± 14.6 (979–1011)	912	1056.8 ± 63.7 (956–1124)	–
Prerectum length	92	87.3 ± 6.7 (82.5–92)	93	98.3 ± 14.4 (89–123)	–
Rectum length	52	51.8 ± 6.6 (47–56)	43	52.7 ± 3.1 (49.5–58)	–
Tail length	26	26.9 ± 1.2 (26–28)	27	28.3 ± 2.5 (26–31)	27.4 ± 3.0 (22–31)
Spicule length	–	–	–		77.4 ± 5.3 (68–83)
Ventromedian supplements	–	–	–		13–15

**Male.** Unknown.

#### Remarks.

This species is very close to the Vietnamese populations identified as *L.glandosum* and described by Vu and Nguyen (2013). It differs only in a slightly wider lip region (22–24 vs 21–22 µm) and somewhat longer odontostyle (35–37 vs 29–35 µm). The odontostyle of the specimens of Cuc Phuong population were measured again and an error was detected in the length of the shortest odontostyle, thus the range of odontostyle length becoming 32–35 µm. The Vietnamese specimens differ from these of the type population of *L.glandosum* by having a longer body (L = 1.6–2.25 vs 1.41–1.58 mm), odontophore (40–50 vs 38–39 µm), pharynx length (437–513 vs 407–420 µm) and tail (25–30 vs 21–24 µm). Besides, the lateral chord is without vs with gland-like structures in *L.glandosum*. Since there are no significant differences based on morphology between the new species and the previously reported Vietnamese populations of *L.glandosum* (Vu and Nguyen 2013), these two populations are considered conspecific.

#### Type locality and habitat.

A pristine mountain area in the Natural Reserve Du Gia, Bac Me District, Ha Giang Province, Vietnam (22°43'5"N, 105°12'4"E, elevation 750 m a.s.l.)

#### Type material.

The holotype female and four paratype females are deposited in the Nematode Collection of the Institute of Biodiversity and Ecosystem Research, Bulgarian Academy of Sciences, Bulgaria, under an accession number IBER-BAS NTC 110.

#### Representative DNA sequences.

After sequencing the obtained *L.porosum* sp. nov. rDNA sequence fragments were deposited in GenBank under the following accession numbers: PP084891 (18s rDNA) originating from NR Bac Me, Ha Giang, Vietnam, and PP060468 (NR Bac Me, Ha Giang, Vietnam), PP060470 (NP Cuc Phuong, Ninh Binh, Vietnam) (28S rDNA).

#### Etymology.

The species name reflects conspicuous ventral and lateral body pores characteristic of the species.

#### Differential diagnosis and relationships.

The new species is characterised by its medium-sized body (1.6–2.25 mm), and odontostyle (32–37 μm), weakly angular and offset by a constriction lip region, 21–24 μm wide, presence of a peculiar cuticular fold on the dorsal side of stoma, not differentiated uterus, a longitudinal vulva (V = 48.5–59%), and short and rounded tail (25–30 μm, c = 53–90, c’ = 0.5–0.6). In having medium body size (L = 1.5–2. 5 mm) and odontostyle (32–39 µm), and lip region offset by a constriction, the new species resembles *L.andrassyi* Gagarin, 1992, *L.brevicauda* Furstenberg, Heyns & Swart, 1993, *L.gerlachi* Andrássy, 2011, *L.glandosum*, and *L.obesum* Thorne, 1974. The new species can be differentiated from all of them by having a peculiar cuticular fold on the dorsal part of the stoma. Further, it differs from:

*L.andrassyi* by having: a somewhat shorter body length (1.6–2.25 vs 2.1–2.7 mm), narrower lip region (21–24 vs 36–38 μm), shorter prerectum and tail (1.6–2.1 vs 3.5–5.4 times longer than anal body diameter, 25–30 vs 35–45 μm respectively) (Gagarin 1992);

*L.brevicauda* by having different shape of amphidial fovea (stirrup vs funnel shape), less robust odontostyle (5–6 vs 4 times as long as broad), absence of disc-shaped structure between pharynx and cardia vs present, smaller vulva (8–10 vs 13 or 14 µm long) stippled area large vs small (Furstenberg et al. 1993);

*L.gerlachi* by having: a somewhat wider lip region (21–24 vs 21–22 μm), more posterior vulva position (48.5–59 vs 45–48%), rectum straight vs angular, a longer prerectum (1.6–2.1 vs 1.3–1.4 anal body widths) and shorter tail (25–30 vs 30–35 μm, c = 53–90 vs 51–53) (Andrássy 2011b);

*L.glandosum* by having: a longer body (1.6–2.25 vs 1.41–1.58 mm), absence of a disc-shaped structure between pharynx and cardia vs present, lateral chord ornamentation (without vs with gland-like structures), wider lip region (21–24 vs 20–21 μm), somewhat longer odontostyle (32–37 vs 32–35 μm) and tail (25–30 vs 21–24 μm) (Rahman et al. 1986);

*L.obesum* by having: weakly vs strongly angular lip region, absence of a disc-shaped structure between pharynx and cardia vs present, tail longer (25–30 vs 37 μm, calculated from the drawing), and without vs with distinctive central core (Thorne 1974).

### ﻿ Labronema bidoupense sp. nov.

Taxon classificationAnimaliaDorylaimidaDorylaimidae

1CBDFD62-1B3D-5F73-B310-88B6ADF8ED8E

https://zoobank.org/60E81CEC-448C-4A08-85BC-A217685F030B

#### Material examined.

Six females, 6 males collected from National Park Bidoup-Nui Ba (Lac Duong District, Lam Dong Province) in good condition.

#### Description.

**Figure 4.**
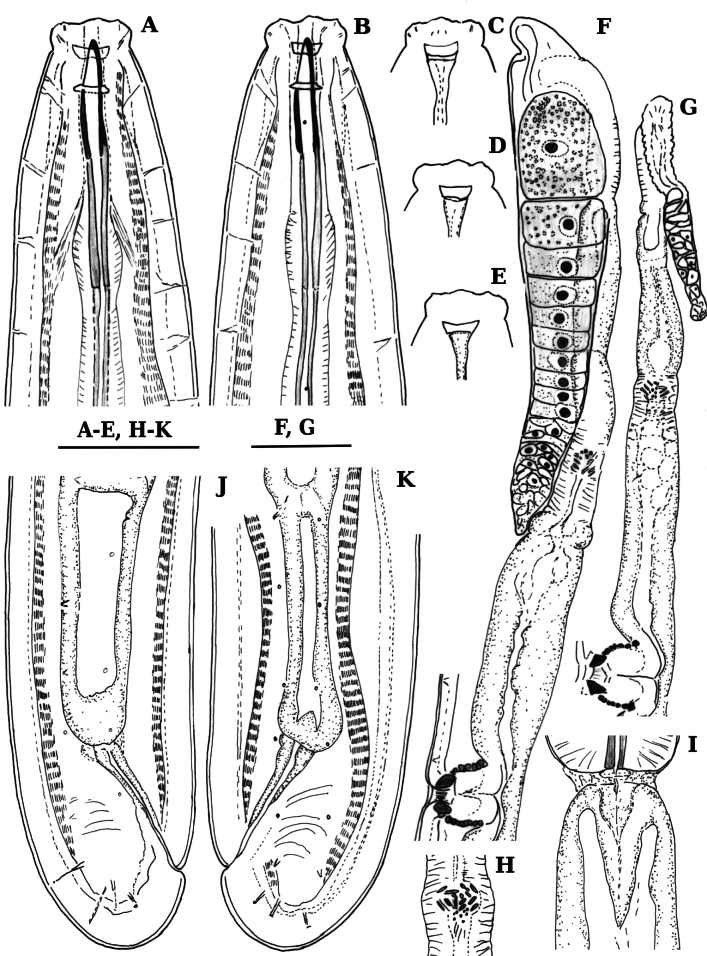
Line drawings of *Labronemabidoupense* sp. nov. female **A, B** lip region (**B** holotype) **C–E** amphids (D holotype) **F, G** anterior genital branch **H** uterine Z-differentiation (holotype) **I** pharyngo-intestinal junction (holotype) **J, K** tail ends (**J** holotype). Scale bars: 25 µm (**A–E, H–K**); 50 µm (**F, G**).

**Figure 5.**
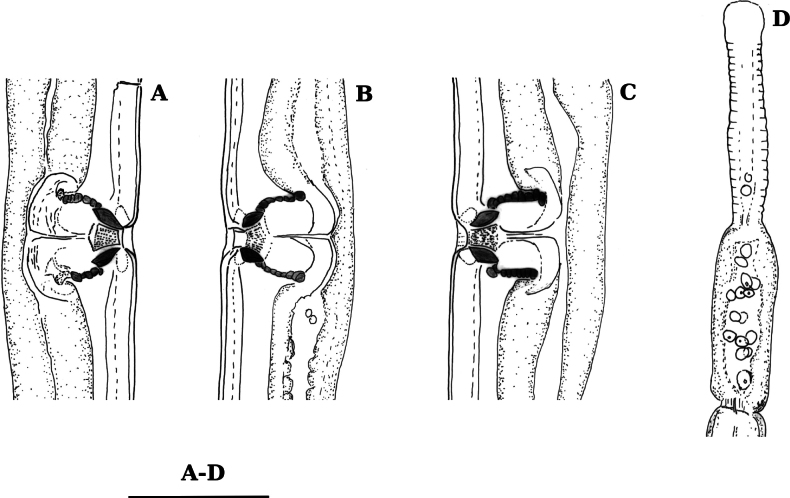
Line drawings of *Labronemabidoupense* sp. nov. female **A–C** vulval regions **D** anterior pars dilatata oviductus Scale bar: 25 µm.

**Figure 6.**
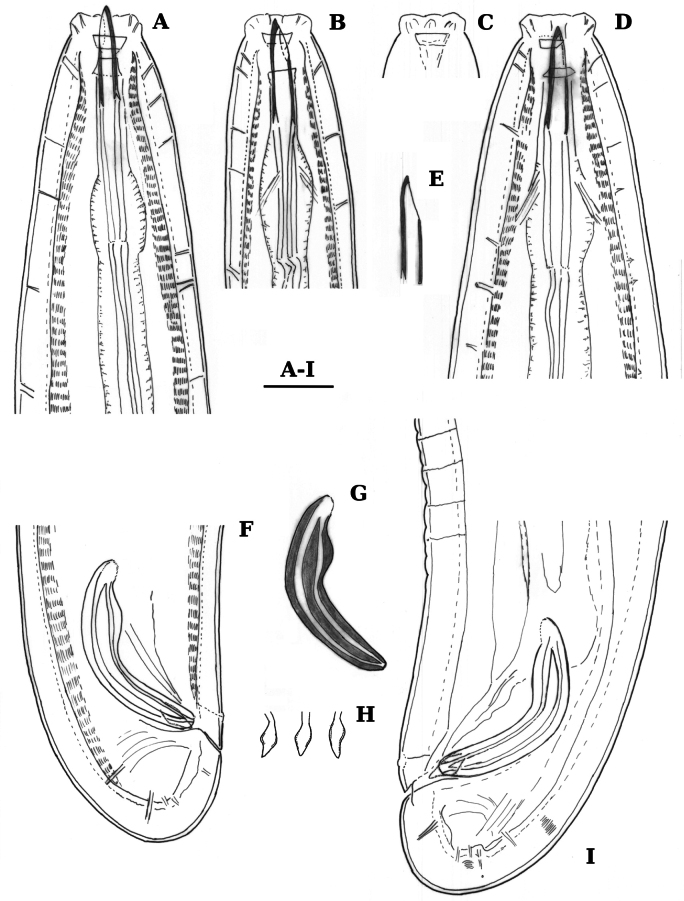
Line drawings of *Labronemabidoupense* sp. nov. male **A, B, D** lip region **C** amphidial fovea **E** odontostyle **F, I** tail ends **G** spicules **H** lateral guiding pieces. Scale bar: 25 µm.

**Figure 7.**
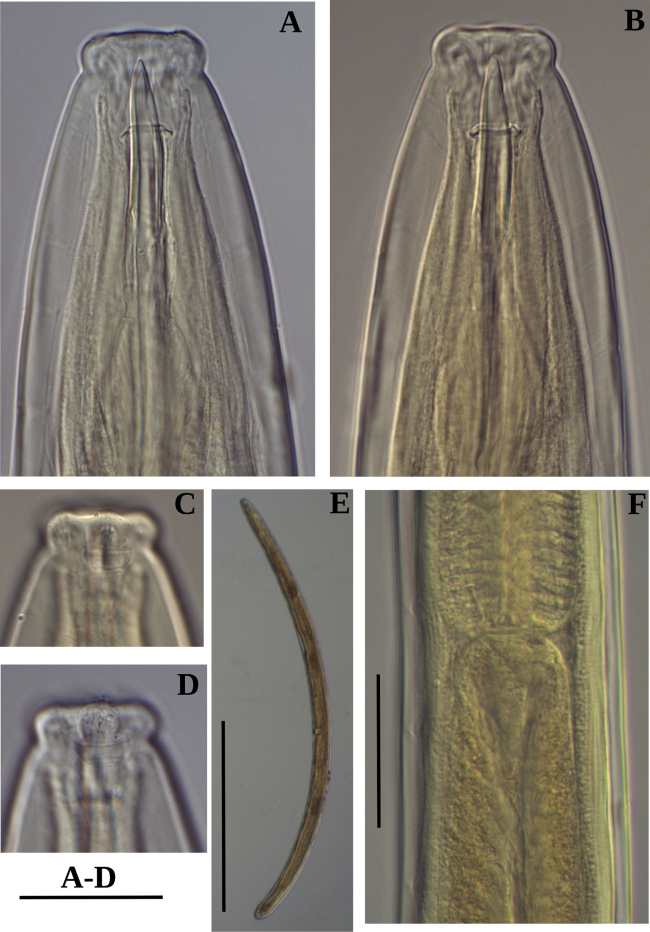
Photomicrographs of *Labronemabidoupense* sp. nov. female **A, B** lip region **C, D** amphidial fovea **E** entire body **F** pharyngo-intestinal junction Scale bars: 30 µm (**A–D**); 50 µm (**F**); 500 µm (**E**).

**Figure 8.**
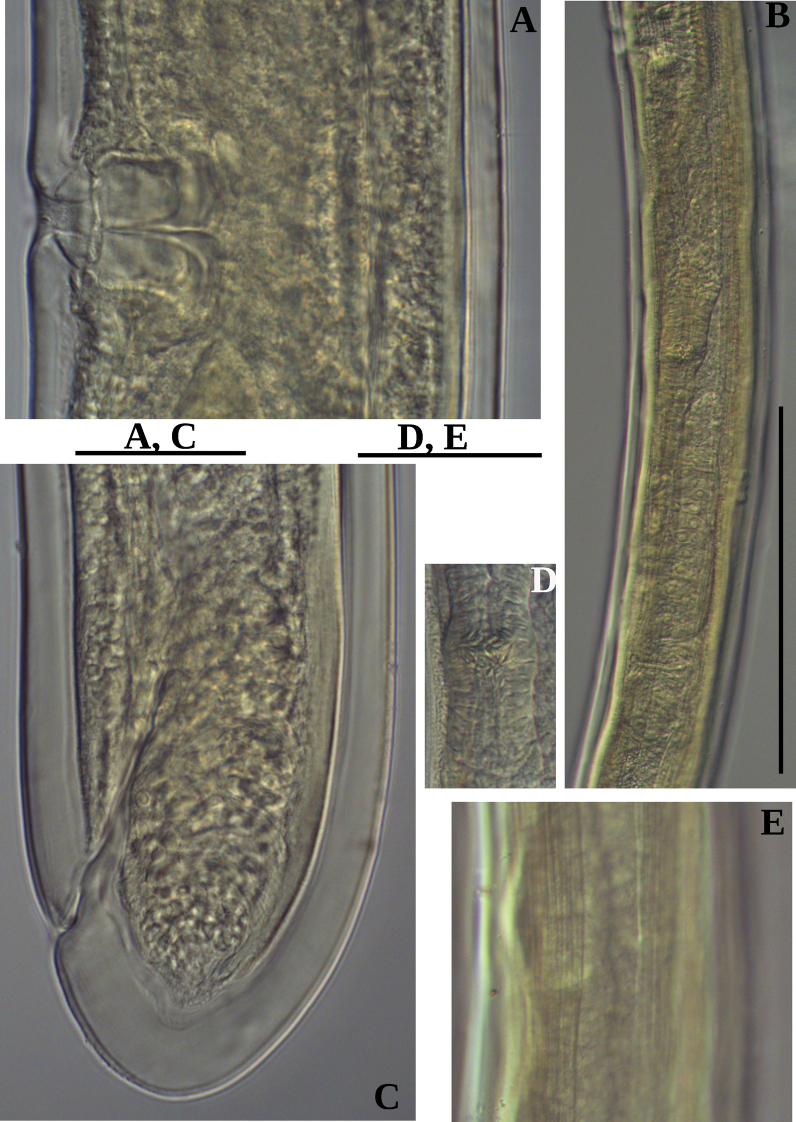
Photomicrographs of *Labronemabidoupense* sp. nov. female **A** vulval region **B** posterior genital branch **C** tail end **D** uterine Z-differentiation **E** lateral field. Scale bars: 30 µm (**A, C**); 200 µm (**B**); 50 µm (**D, E**).

**Figure 9.**
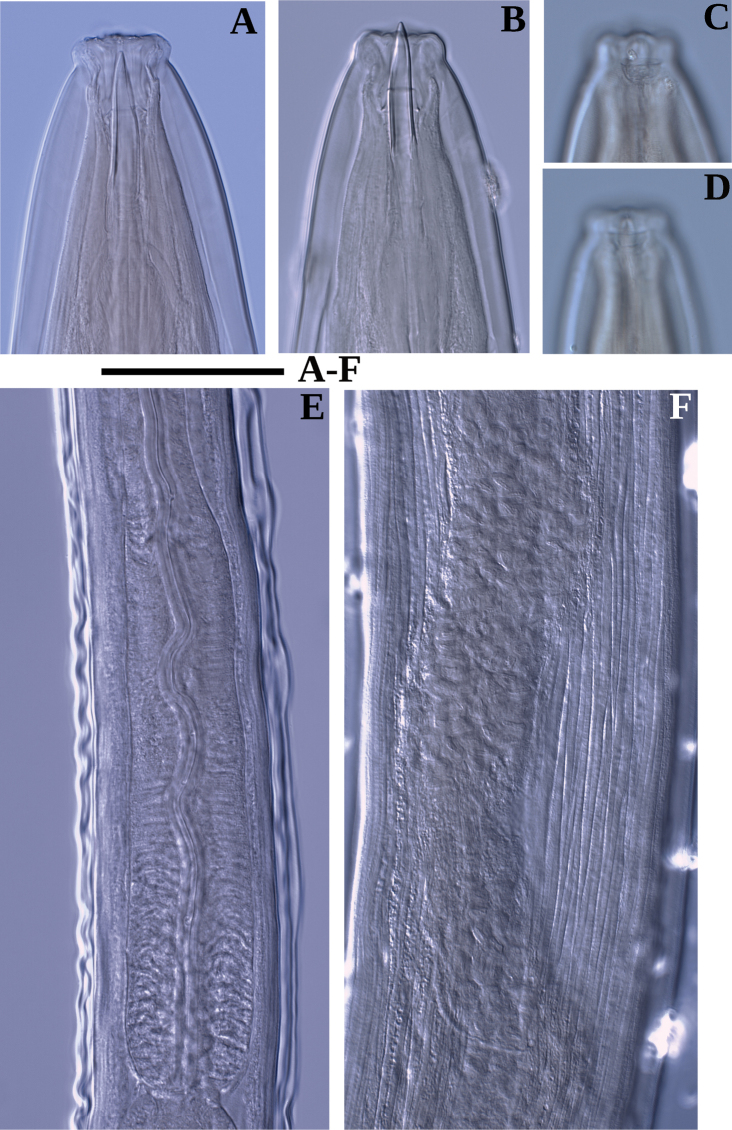
Photomicrographs of *Labronemabidoupense* sp. nov. male **A, B** lip region **C, D** amphidial fovea **E** pharyngeal expansion **F** sperm cells in testis. Scale bar: 50 µm.

**Figure 10.**
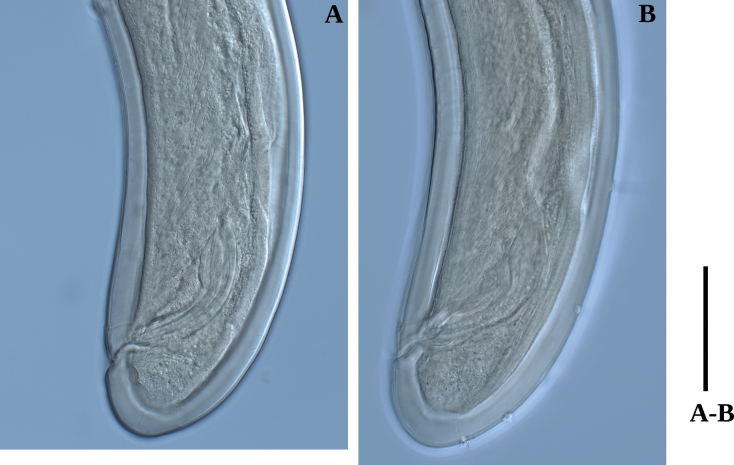
Photomicrographs of *Labronemabidoupense* sp. nov. male **A, B** tail ends. Scale bar: 50 µm.

**Females** (for measurements see Table 1, Figs 4–10) Nematodes of a medium size. Body cylindrical, slightly curved ventrally or adopted an open C shape after fixation. Cuticle three layered, especially obvious at caudal region, outer layer thin, intermediate layer much thicker than the outer one, particularly at the caudal region; inner layer much thinner than the intermediate layer; cuticle 7–8 μm thick at anterior region, 6–7 μm in mid-body, and 12–14 μm posterior to anus. The intermediate layer with longitudinal striations seen at a certain optical section. A narrow cervical lacuna between cuticle and epidermis observed. Ventral and lateral pores conspicuous, located all over the body, dorsal pores four or five at anterior end. After fifth or sixth ventral pore a structure resembling an excretory pore (duct with cuticularised walls) present (at 98–107 μm from anterior end). Lip region truncated, laterally somewhat angular, offset by a deep constriction, 3.2–3.6 times broader than high, less than one-third (33–38%) of body diameter at pharynx base. Lips weakly separated, labial and cephalic papillae very low, liplets around labial opening present (Fig. 9B). Amphid with stirrup-shaped fovea; its aperture 11 μm wide, occupying two-fifths to one-half of lip region diameter. Cheilostom nearly cylindrical, with thick walls. Odontostyle strong and slightly sigmoid, 6–7 times as long as broad, 1.4–1.5 times longer than lip region diameter, and 1.9–2.4% of total body length. Odontophore rod-like; 1.1 times odontostyle length. Pharynx strongly muscular, with its slender portion enlarging gradually, basal expansion 203–222 μm long, 43–46% of pharynx length; gland nuclei and their orifices located as follows: DO = 52%, DN = 57%, S_2_N = 93% (*n* = 1). Pharyngo-intestinal junction well developed; cardia with long conical projection into the intestinal lumen measuring 50–65 × 17–24 µm; presence of a thin and irregular disc- or belt-like structure separating the pharyngeal base from the cardia. Genital system di-ovarian, with both branches well and equally developed. Ovaries are reflexed, anterior 204–261 μm long, posterior 208–262 μm long, reaching oviduct/uterus junction, with oocytes first in two or more rows and then in a single row. Anterior oviduct 92.5–149 μm long and posterior oviduct 126–154 μm long, respectively, or 1.35–1.84× longer than body diameter, consisting of a moderately developed proximal *pars dilatata*. *Pars dilatata* elongated and measuring 56–80 × 17–21 μm (27–33 × 16 μm in one young female), often containing round spermatozoa. Uterus complex, tripartite, anterior uterus 195–244 μm long, posterior uterus 193–241 μm long or 2.4–2.8 times longer than the body diameter; consisting of a thicker proximal region with lumen, a muscularised region (*pars musculosa uteri*) with Z-differentiation (Fig. 4F–H), ending with a tubular part. In the young female, the measurements are as follows: anterior ovary, oviduct, and uterus 106, 65, and 190 μm long, respectively, and posterior ovary, oviduct, and uterus 102, 65, and 217 μm long, respectively. Vagina extending inwards to 44% of the body diameter: *pars proximalis* 22–29 × 19–26 μm in size, *pars refringens* consisting of (lateral view) two small triangular sclerotized pieces, with a combined width of 18–22 μm; *pars distalis* 9 μm long. Vulva a longitudinal slit 7–9 μm long. Prerectum 1.7–2.4 and rectum 1–1.2 times longer than anal body diameter. Tail short and rounded, three pairs of caudal pores at the posterior half of the tail. Hyaline part of tail 12–14 μm thick or 42–53% of total tail length.

**Males.** General morphology similar to that of the female, except for the genital system. After the fourth or fifth ventral pore a structure resembling an excretory pore (duct with cuticularised walls) present (at 96–105 μm from anterior end). Genital system di-orchic, composed of two opposed testes, anterior 207–234 μm and posterior 191–225 μm long. Sperm oval, measuring 5 μm. Ventromedian supplements contiguous 13–15 in number, ad-cloacal pair located at 9–13 μm from cloacal aperture. Spicules 1.4–1.8 times body diameter at cloacal aperture long, 5–6 times as long as wide, spicule head 0.8–1.4 times longer than wide, occupying 9–11% of total spicule length, and with slender walls, median piece narrow, occupying 29–36% of spicule maximum width, and reaching the posterior end, which is 5–6 μm broad, curvature 140–150°, ventral hump located at 20–23 μm or 23.5–30% of spicule length from its anterior end, posterior tip 4.5–6 μm wide. Lateral guiding piece slightly curved, leaf-shaped (Fig. 6H), 17–19 μm long and 6 µm wide, ca 3 times longer than broad. Tail similar to that of female.

#### Type locality and habitat.

A pristine forest in the Bidoup mountain, Lac Duong District, Lam Dong Province, Vietnam (12°04'47"N, 108°39'29"E, elevation 2130 m a.s.l.).

#### Type material.

The holotype female, three paratype females, and two paratype males are deposited in the Nematode Collection of the Institute of Biodiversity and Ecosystem Research, Bulgarian Academy of Sciences, Bulgaria, under the accession numbers IBER-BAS NTC 111, 112, respectively. One paratype female and four paratype males are deposited in the Nematode collection of the Institute of Ecology and Biological Resources, Hanoi, Vietnam (accession number IEBR-FLN-DOR_04 and 05–08, respectively), one paratype female is deposited in the Wageningen Nematode Collection (**WANECO**), Wageningen, the Netherlands (WANECO accession number WT 4040), and one paratype female is deposited in the Nematode Collection of the U.S. Department of Agriculture (**USDA**), Beltsville, Maryland, USA (USDANC accession number T-8110p).

#### Representative DNA sequences.

After sequencing the obtained *L.bidoupense* sp. nov. rDNA sequence fragments were deposited in GenBank under the following accession numbers PP084892 (18S rDNA) and PP060469 (28S rDNA), both originating from a specimen collected in NP Bidoup-NuiBa, Dak Lak, Vietnam.

#### Etymology.

The species is named after the Bidoup Mountain, the place from where it was recovered.

#### Differential diagnosis and relationships.

The new species is characterised by its medium-sizes body (1.59–2.04 mm long), lip region offset by a deep constriction and 25–28 μm wide, odontostyle 37.5–39 μm long, uterus complex (tripartite), longitudinal vulva (V = 54–57%), short and rounded tail (26–31 μm, c = 57.8–77.2, c’ = 0.5–0.6). Males with 68–83 μm long spicules, 5–6 times as long as wide and contiguous ventromedian supplements 13–15 in number, lateral piece leaf-shaped.

In having medium body size (L = 1.5–2.5 mm) and odontostyle (31–39 μm), and lip region offset by a constriction, the new species resembles *L.andrassyi*, *L.brevicauda*, *L.gerlachi*, *L.glandosum*, *L.obesum* and *L.porosum* sp. nov. The new species differs from:

*L.porosum* sp. nov. by having: a thicker body cuticle, wider lip region (25–28 vs 21–24 μm), slightly sigmoid vs straight odontostyle, longer odontostyle (37.5–39 vs 32–37 μm), a peculiar cuticular fold on the dorsal site of stoma absent vs present, shorter pharyngeal expansion (203–222 vs 220–237 μm), presence of disc-shaped structure between pharynx and cardia vs absence, complex vs simple uterus, males present vs absent;

*L.andrassyi* by having: a shorter body length (1.59–2.04 vs 2.1–2.7 mm), thicker body cuticle, narrower lip region (25–28 vs 36–38 μm), longer prerectum (1.7–2.4 vs 3.5–5.4 times longer than anal body diameter) and tail (26–31 vs 35–45 μm), males present vs absent (Gagarin 1992);

*L.brevicauda* by having: a different number of dorsal pores at anterior end (4 or 5 vs 6 or 8), wider lip region (25–28 vs 21–25 μm), longer and less robust odontostyle (37.5–39 vs 32–35.6 μm and 6–7 vs 4 times as long as broad), complex vs simple uterus, males present vs absent (Furstenberg et al. 1993);

*L.gerlachi* by having: a wider lip region (25–28 vs 21–22 μm), longer odontostyle (37.5–39 vs 33–35 μm), more posterior vulva position (54–57 vs 45–48%), rectum straight vs angular, shorter tail (26–28 vs 30–35 μm), males present vs absent (Andrássy 2011b);

*L.glandosum* by having: a differently shaped lateral chord (without vs with gland-like structures), a wider lip region (25–28 vs 20–21 μm), longer odontostyle (37.5–39 vs 32–35 μm) and tail (26–31 vs 21–24 μm), complex vs simple uterus, males present vs absent (Rahman et al. 1986);

*L.obesum* by having: a shorter body length (1.59–2.04 vs 2.2 mm), longer odontostyle (37.5–39 vs 33 μm), tail without vs with distinctive central core, males present vs absent (Thorne 1974).

##### ﻿Sequences and phylogenetic analyses

**Figure 11.**
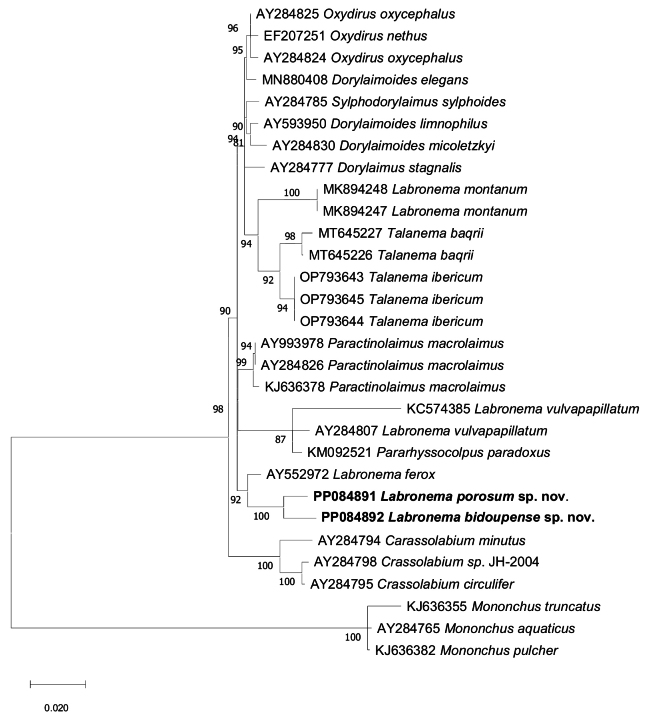
Phylogenetic relationships of *Labronema* from the analysis of the 18S rDNA sequences under ML (K2+G+I model). Numbers to the left of the branches are bootstrap values for 1000 replications.

**Figure 12.**
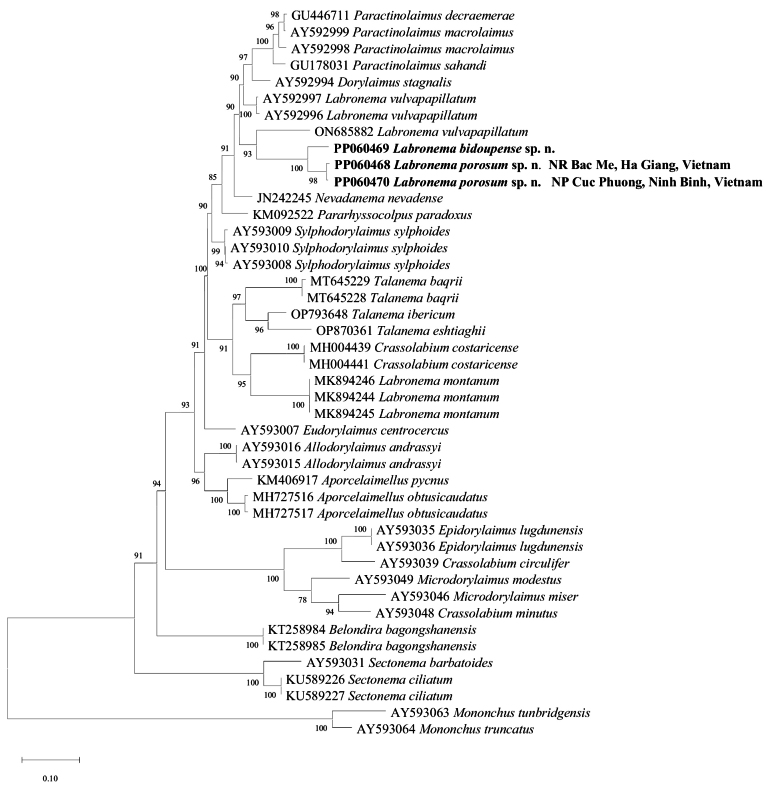
Phylogenetic relationships of *Labronema* from the analysis of the 28S rDNA sequences under ML (K2+G+I model). Numbers to the left of the branches are bootstrap values for 1000 replications.

Molecular sequences of two specimens of *L.porosum* sp. nov. and one specimen of *L.bidoupense* sp. nov. were analysed in this study. After sequencing and editing, five sequences were obtained: a nearly full-length of 18S rRNA gene for *L.porosum* sp. nov., (1641 bp; PP084891) and *L.bidoupense* sp. nov., (1636 bp; GenBank: PP084892); two nearly full-length D2-D3 segment of 28S rRNA gene for *L.porosum* sp. nov., (828 bp; GenBank: PP060468, PP060470) and for *L.bidoupense* sp. nov. (856 bp; GenBank: PP060469). A BLAST search for matches to the partial 18S rDNA sequences revealed that *L.porosum* sp. nov. has a difference of 34 nt with *L.ferox* (AY552972), 24–40 nt with *L.vulvapapillatum* (AY284807 from The Netherlands, KC574385 from Iran), 32 nt with *L.montanum* (MK894247–MK894248) and 23 nt with the new species *L.bidoupense* sp. nov. The new species *L.bidoupense* sp. nov. has a difference of 34 nt with *L.ferox* (AY552972), 22–69 nt with *L.vulvapapillatum* (AY284807 from The Netherlands, KC574385 from Iran) and 36 nt with *L.montanum* (MK894247–MK894248). A BLAST search for matches the partial 28S rDNA sequences revealed that *L.porosum* sp. nov. has a difference of 76–78 nt with *L.vulvapapillatum* (AY592996–АY592997 from The Netherlands, ON685882 from Iran), 106 nt with *L.montanum* (MK894244–MK894246) and 31 nt with *L.bidoupense* sp. nov. *Labronemabidoupense* sp. nov. has 78 nt of difference with *L.vulvapapillatum* (AY592996–AY592997 from The Netherlands, ON685882 from Iran), 115 nt with *L.montanum* (MK894244–MK894246) from Spain. *Labronemaporosum* sp. nov. population from Du Gia Nature Reserve, Bac Me district, Ha Giang Province (PP060468) was 100% identical with *Labronema* population from Cuc Phuong National Park, Ninh Binh Province (PP060470). The evolutionary relationships of the two new species as derived from the molecular analyses, are presented in the phylogenetic trees (Figs 11, 12). The 18S rDNA sequences of the two studied *Labronema* species from Vietnam clustered together in a group with *L.ferox* and were nested within the first clade (following Shokoohi et al. 2013), encompassing representatives of other species of *Labronema* (*L.vulvapapillatum*) and genera *Paractinolaimus* and *Pararhyssocolpus* (Fig. 11). Generally, this tree topology positioning was confirmed by the phylogenetic analyses based on the 28S rDNA data (Fig. 12). The 28S rDNA sequences of the two new species from Vietnam clustered together with *L.vulvapapillatum* from Iran (ON685882) and were positioned within a clade containing the genera belonging to the family Dorylaimidae (following the Peña-Santiago and Abolafia 2019 and Vazifeh et al. 2023) and encompassing representatives of the genera *Crassolabium*, *Nevadanema*, and *Dorylaimus*, as well as genera from other families *Talanema* (Qudsianematidae), *Pararhyssocolpus* (Pararhyssocolpidae), *Sylphodorylaimus* (Thornenematidae), and *Paractinolaimus* (Actinolaimidae). The close relationships of both Vietnamese species are also in agreement with their morphology. The main differences were described in detail in the diagnosis part, in the description of *L.bidoupense* sp. nov.

The most recent identification keys of all *Labronema* species known by that time is the key by Andrássy (1991). The same author also provided a key to the European species of the genus (Andrássy 2009). However, these keys are outdated due to the numerous taxonomic changes and several new species described during the last decades. Here we elaborated a partial key for determination of a group of species with a medium-sized odontostyle (31–39 μm) to which also belong the two new Vietnamese species. Some species are excluded from the key:

- *Labronemavirgo* Monteiro 1970: this species fits the features of the genus *Labronema* (the presence of offset by constriction lip region, robust odontostyle 1.5 times lip region diameter, double guiding ring), but because of the transverse vulva non-typical for the genus, this species is not included in the key.
- *Labronemaenigmatum* Baniyamuddin & Ahmad, 2007: in the presence of a transverse vulva, spaced ventromedian supplements, and bluntly rounded tail, this species does not fit well with typical *Labronema* pattern. Andrássy (2009) transferred this species to the genus *Labronemella* Andrássy, 1985. According to Peña-Santiago et al. (2012), the identity of this species is questionable; therefore, it should be either considered a species *inquirenda* or retained under *Labronema*. Because of the problematic position of this species, it is not included in the key.
- *Labronemadiversum* Andrássy, 2002: this species is characterised by sexual dimorphism in the tail region, females have a tail with a dorsally curved peg, while males have a rounded conoid tail. The sexual dimorphism is atypical for the genus *Labronema*. According to Andrássy (2011a) this peculiar structure of the female tail is an atavistic character. The presence of robust odontostyle, double guiding ring, longitudinal vulva and contiguous ventromedian supplements fit with genus *Labronema* but its position is doubtful (Imran et al. 2021).
- *Labronemanemella* Mushtaq & Ahmad, 2007: according to Peña-Santiago et al. (2012) the true identity of this species is intriguing, because of the morphology of lip region and odontostyle (our remark). The authors consider that this species might be a member of *Labronemella*, but further data is needed to confirm this.
- Two of the atypical species of genus *Labronema* (characterised with lip region nearly continuous with the adjacent body) – *L.neopacificum* Rahman, Jairajpuri, Ahmad & Ahmad, 1986 and *L.pacificum* (Cobb, 1906) Thorne, 1939, which are distinguished by a medium-sized odontostyle are not included in this key because the lip region does not correspond to the typical offset lip region of members of the genus *Labronema.*

### ﻿Key to the species of genus *Labronema* with medium sized odontostyle (31–39 µm)

**Table:** 

1	Body large, 3 mm or more	**2**
–	Body < 3 mm	**5**
2	Vulva pre-equatorial – ♀ L = 3.4–3.6 mm, a = 45.3–46.03, b = 4.7–4.8, c = 109.8–113.2, V = 47–49% **(India)**	***L.mannai* Dattaray, Roy, Gantait, 2015**
–	Vulva equatorial	**3**
3	Odontostyle nearly equal to lip region diameter, males present – ♀ L = 3.0–3.6 mm, a = 35, b = 4.1, c = 100, V = 50%, ♂ L = 3.0–3.5 mm, a = 43, b = 4.0–5.2, c = 100, ventromedian supplements 20–27	***L.ferox* Thorne, 1939 (USA)**
–	Odontostyle longer than lip region diameter, males absent	**4**
4	Prerectum longer, 3–4 times anal body diameter, paravulvae present anterior and posterior to vulva – ♀ L = 3.2–4.0 mm, a = 45–50, b = 4.3–5.4, c = 119–154, V = 49–56% **(India)**	***L.deoriaensis* Khan, Jairajpuri & Ahmad, 1989**
–	Prerectum shorter, twice as long as anal body diameter, paravulvae absent – ♀ L = 3.0–3.7 mm, a = 37–44, b = 3.8–4.9, c = 90–127, V = 51–54% (USA)^*^	***L.thornei* Ferris, 1968**
5	Lip region off set by weak depression – ♀ L = 2.4–3.0 mm, a = 28–33, b = 4.3–5.0, c = 90–103, V = 44–49%, ♂ L = 2.20–2.76 mm, a = 33–38, b = 3.9–4.5, c = 74–93, ventromedian supplements 21–23 **(Ecuador)**	***L.aequatoriale* Andrássy, 2011**
–	Lip region off set by constriction	**6**
6	Body length 2.3–3 mm; lip region diameter as long as odontostyle length – ♀ L = 2.1–2.8 mm, a = 19.5–28, b = 4.0–4.3, c = 54.7–73.7, V = 51–57% (**Russia)**	*L.andrassyi* Gagarin, 1992 ^*^
–	Body length < 2.2 mm, lip region diameter shorter than odontostyle length	**7**
7	Vulva pre-equatorial – ♀ L = 1.7–2.0 mm, a = 17–19, b = 3.7–4.0, c = 51–53, V = 45–48% **(Seychelles)**	***L.gerlachi* Andrássy, 2011**
–	Vulva equatorial	**8**
8	Lateral chords with gland-like structures – ♀ L = 1.4–1.6 mm, a = 19–22, b = 3.4–3.7, c = 59–71, V = 53–54% **(India)**	***L.glandosum* Rahman, Jairajpuri, Ahmad & Ahmad, 1986**
–	Lateral chords without gland-like structures	**9**
9	Tail with distinctive central core – ♀ L = 2.2 mm, a = 25, b = 4.1, c = 60, V = 53% **(USA)**	***L.obesum* Thorne, 1974**
–	Tail without central core	**10**
10	Female genital system tripartite, males present – ♀ L = 1.6–2.0 mm, a = 20.9–23.4, b = 3.6–4.1, c = 57.8–77.2, V = 54–57%, ♂ L = 1.4–2.0 mm, a = 19.7–25, b = 3.6–4.5, c = 58.8–67.8, ventromedian supplements 13–15 in number **(Vietnam)**	***L.bidoupense* sp. nov.**
–	Female genital system simple, males absent	**11**
11	The disc-like structure between the pharynx base and cardia present – ♀ L = 1.6–2.1 mm, a = 21.9–23, b = 3.7–4.0, c = 64–79, V = 50–52% **(Madagascar)**	***L.brevicauda* Furstenberg, Heyns & Swart, 1993**
–	The disc-like structure between the pharynx base and cardia absent – ♀ L = 1.6–2.25 mm, a = 18–25, b = 3.5–4.6, c = 53–90, V = 49–59% **(Vietnam)**	***L.porosum* sp. nov.**

## ﻿Discussion

The integrative taxonomy approach used in the study of *L.porosum* sp. nov. and *L.bidoupense* sp. nov. from Vietnam contributes to the knowledge of the large and complex genus *Labronema*. The two species are very close morphologically and genetically and form a well-supported clade with the type species (18S rDNA tree Fig. 11) and *L.vulvapapillatum*, Vakil Kandi population from Iran (28S rDNA tree Fig. 12). Other populations identified as *L.vulvapapillatum* from The Netherlands (AY284807) and Kerman Province, Iran (KC574385) clustered together in the 18S r DNA tree (Fig. 11) and are closely related to that clade (Fig. 11). Obviously, the populations of *L.vulvapapillatum* from The Netherlands and Kerman Province, Iran are not conspecific with that from Vakil Kandi population. This species has been reported from several countries in Europe and Asia (Shokoohi et al. 2013; Zahedi Asl. et al. 2023) and since the ranges for many characters (e.g., body, odontostyle, and tail length) are very wide (Shokoohi et al. 2013: table 5), most probably it represents a species complex (Shokoohi et al. 2013). However, it is difficult to delineate different species based on literature sources because in some descriptions data about reproductive system or illustrations of important characters are missing or it is difficult to be assessed from the photomicrographs.

In both phylogeny trees of 18S and 28S rDNA (Figs 11, 12), *L.montanum* stands independently with other species of *Labronema* (*L.ferox*, *L.vulvapapillatum* and the two new species from Vietnam) and it is closer to the species of *Talanema* and *Crassolabiumcostaricense* Varela-Benavides & Peña-Santiago, 2018. *Labronemamontanum* is very similar to other three *Labronema* species: *L.angeloi*, *L.carussoi* (Vinciguerra an Orselli 1998) and *L.duxousi* (= *L.pulchrum*) (Peña-Santiago and Vinciguerra 2019). As it was mentioned by Peña-Santiago and Abolafia (2019) that these four species represent a rather homogeneous group. They possess unique combination of several characters such as lip region shape, presence of structures resembling cardial glands, short conical cardia, and peculiar reproductive system in female – tripartite uterus with narrow often coiled intermediate part, *pars refringens vaginae* of similar appearance; and in males – the lateral piece is narrow and bifid. These four species have distinct range inhabiting the southern Europe and have been reported from France, Albania, Spain, Italy, Switzerland, and Romania (Peña-Santiago and Abolafia 2019).

The 28S rDNA sequences from the two populations of *Labronema* from Du Gia Nature Reserve (Bac Me District, Ha Giang Province) and Cuc Phuong National Park (Ninh Binh Province) confirmed that the population previously recorded as *L.glandosum* from Cuc Phuong National Park (Vu and Nguyen 2013) is identical with the new species *L.porosum* sp. nov.

It is very difficult to assess the phylogenetic relationships of the genus *Labronema* with other genera using the integrative approach due to the very low number of species studied, also affecting the reconstruction of the evolutionary history of dorylaimid groups of higher taxonomic ranks.

## Supplementary Material

XML Treatment for Labronema porosum

XML Treatment for Labronema bidoupense

## Footnotes

In the original description, no data on vulva characterisation were presented.

## ﻿Additional information

### Author ORCIDs

Tam T. T. Vu https://orcid.org/0000-0003-1145-975X

Milka Elshishka https://orcid.org/0009-0002-3588-4566

Anh D. Nguyen https://orcid.org/0000-0001-9273-0040

### Data availability

All of the data that support the findings of this study are available in the main text or Supplementary Information.

## Supplementary materials

Supplementary material 1 An updated list of species of genus *Labronema* Thorne, 1939 Data type docx https://binary.pensoft.net/file/1152236This dataset is made available under the Open Database License (http://opendatacommons.org/licenses/odbl/1.0/). The Open Database License (ODbL) is a license agreement intended to allow users to freely share, modify, and use this Dataset while maintaining this same freedom for others, provided that the original source and author(s) are credited.Tam T. T. Vu, Milka Elshishka, Anh D. Nguyen, Thi Mai Linh Le, Aleksandar Mladenov, Vlada Peneva
