# Supplementary material for: ﻿Two new species of genus Labronema Thorne, 1939 (Nematoda, Dorylaimidae) from natural parks of Vietnam with an identification key to the species with a medium-sized odontostyle
Source: Zookeys. 2024 Oct 11;1215:1–26. doi: 10.3897/zookeys.1215.128183 (PMC11489713; doi:10.3897/zookeys.1215.128183)
Supplement: Supplementary material 1 — An updated list of species of genus Labronema Thorne, 1939 [file zookeys-1215-001_article-128183__-s001.docx]

**An updated list of species of genus *Labronema* Thorne, 1939**

**February 2024**

1. *Labronema aequatoriale* Andrássy, 2011
2. *Labronema alticolum* (Menzel in Hofmänner & Menzel, 1914) Thorne, 1939
3. *Labronema andrassyi* Gagarin, 1992
4. *Labronema angeloi* Vinciguerra & Clausi, 1994
5. *Labronema arenicola* (Altherr, 1958) Andrássy, 1986
6. *Labronema bathybium* (Daday, 1906) Andrássy, 1960
7. *Labronema bicuticulum* Furstenberg, Heyns & Swart, 1993
8. *Labronema brevicauda* Furstenberg, Heyns & Swart, 1993
9. *Labronema carusoi* Vinciguerra & Orselli, 1998
10. *Labronema chilense* Andrássy, 1967
11. *Labronema confusum* (Jana & Baqri, 1983) Andrássy, 1992
12. *Labronema corii* (Lieberman, 1928) Andrássy, 1960
13. *Labronema deoriaense* Khan, Jairajpuri & Ahmad, 1989
14. *Labronema distinctum* Álvarez-Ortega, Vu & Peña-Santiago, 2010
15. *Labronema diversum* Andrássy, 2002
16. *Labronema duhouxi* (Altherr, 1963) Álvarez-Ortega & Peña-Santiago, 2013
17. *Labronema enigmatum* Baniyamuddin & Ahmad, 2007
18. *Labronema ferox* Thorne, 1939
19. *Labronema gerlachi* Andrássy, 2011
20. *Labronema glandosum* Rahman, Jairajpuri, Ahmad & Ahmad, 1987
21. *Labronema goodeyi* Altherr & Delamare-Deboutteville, 1972
22. *Labronema hyalinum* (Thorne & Swanger, 1936) Thorne, 1939
23. *Labronema khazariense* (Tchesunov, 1985) Andrássy, 1992
24. *Labronema korandum* Choi, Khan & Choi, 2001
25. *Labronema latum* (Cobb, 1891) Andrássy, 1986
26. *Labronema magnum* (Altherr, 1972) Peña-Santiago, 2022
27. *Labronema mangalorense* Ahmad & Ahmad, 2003
28. *Labronema mannai* Dattaray, Roy & Gantait, 2015
29. *Labronema minimum* Dattaray, Roy & Gantait, 2015
30. *Labronema montanum* Peña-Santiago & Abolafia, 2019
31. *Labronema nemellum* Mushtaq & Ahmad, 2007
32. *Labronema neopacificum* Rahman, Jairajpuri, Ahmad & Ahmad, 1987
33. *Labronema nepalense* Ahmad & Jairajpuri, 1982
34. *Labronema obesum* Thorne, 1974
35. *Labronema octodurense* Altherr, 1950
36. *Labronema orientale* Andrássy, 2011
37. *Labronema pacificum* (Cobb, 1906) Thorne, 1939
38. *Labronema papillatum* Khan, Ahmad & Jairajpuri, 1995
39. *Labronema rapax* Thorne, 1974
40. *Labronema rikia* Yeates, 1967
41. *Labronema seychellense* Furstenberg, Heyns & Swart, 1993
42. *Labronema shingalese* Andrássy, 2011
43. *Labronema stechlinense* Altherr, 1968
44. *Labronema thornei* Ferris, 1968
45. *Labronema varicaudatum* (Thorne, 1929) Thorne, 1939
46. *Labronema virgo* Monteiro, 1970
47. *Labronema vulvapapillatum* (Meyl, 1954) Loof & Grootaert, 1981
